# How the Presence of Crystalline Phase Affects Structural Relaxation in Molecular Liquids: The Case of Amorphous Indomethacin

**DOI:** 10.3390/ijms242216275

**Published:** 2023-11-13

**Authors:** Roman Svoboda, Marek Pakosta, Petr Doležel

**Affiliations:** 1 Department of Physical Chemistry, Faculty of Chemical Technology, University of Pardubice, Studentská 573, 532 10 Pardubice, Czech Republic; 2 Faculty of Electrical Engineering and Informatics, University of Pardubice, nam. Cs. legii 565, 530 02 Pardubice, Czech Republic; marek.pakosta@student.upce.cz (M.P.); petr.dolezel@upce.cz (P.D.)

**Keywords:** indomethacin, structural relaxation, crystallinity, TNM model

## Abstract

The influence of partial crystallinity on the structural relaxation behavior of low-molecular organic glasses is, contrary to, e.g., polymeric materials, a largely unexplored territory. In the present study, differential scanning calorimetry was used to prepare a series of amorphous indomethacin powders crystallized to various extents. The preparations stemmed from the two distinct particle size fractions: 50–125 µm and 300–500 µm. The structural relaxation data from the cyclic calorimetric measurements were described in terms of the phenomenological Tool–Narayanaswamy–Moynihan model. For the 300–500 µm powder, the crystalline phase forming dominantly on the surface led to a monotonous decrease in the glass transition by ~6 °C in the 0–70% crystallinity range. The activation energy of the relaxation motions and the degree of heterogeneity within the relaxing matrix were not influenced by the increasing crystallinity, while the interconnectivity slightly increased. This behavior was attributed to the release of the quenched-in stresses and to the consequent slight increase in the structural interconnectivity. For the 50–125 µm powder, distinctly different relaxation dynamics were observed. This leads to a conclusion that the crystalline phase grows throughout the bulk glassy matrix along the internal micro-cracks. At higher crystallinity, a sharp increase in T_g_, an increase in interconnectivity, and an increase in the variability of structural units engaged in the relaxation motions were observed.

## 1. Introduction

The glass transition is a fundamentally essential phenomenon that characterizes the behavior of all amorphous materials [1,2]. Below the glass transition, the structure of the material is solidified (hard and brittle), with practically no diffusion or viscous flow taking place. Above the glass transition, a liquid-like structure dominates in the material, which, as a consequence, becomes more reactive, flexible, and pliant. Depending on the material class (inorganic glasses, polymers, molecular glasses, etc.), the material’s macroscopic mechanistic behavior in the liquid-like temperature region can exhibit either softening, flow, or a rubbery state [3,4,5]. Similar to the mechanical properties (strength, elasticity, brittleness, and deformation behavior), the glass transition manifests itself through large changes in numerous other properties: density, thermal expansion coefficient, heat capacity, electrical conductivity and dielectric constant, transparency, or molecular mobility [6,7,8]. Vast numbers of different glassy, polymeric, and generally amorphous materials are being utilized in modern hi-tech applications, as well as throughout everyday common manufacturing/life. Hence, the exploration of the glass transition (be it theoretical or through the determination of the characteristic temperature T_g_ associated with this phenomenon) stands in the spotlight of the materials and solid-state sciences [9,10,11,12,13]. Note that a simple search within the Web of Science database (keyword “glass transition”) generates over 53,000 entries in just the last 10 years. 

The underlying process that defines the rate and magnitude of the changes of the above-mentioned properties at the glass transition is called structural relaxation [14,15]. The kinetics of the structural relaxation movements are nowadays commonly described in terms of the phenomenological Tool–Narayanaswamy–Moynihan (TNM) relaxation model [16,17,18]:

(1) $$\Phi (t)=\exp\left[-{\left(\int_{0}^{t}\frac{\mathrm{dt}}{\tau \left({T,T}_{f}\right)}\right)}^{\beta }\right]$$

(2) $${\tau (T,T}_{f})=A_{\mathrm{TNM}}\cdot \exp\left[x\frac{{\Delta h}^{*}}{\mathrm{RT}}+(1-x)\frac{{\Delta h}^{*}}{{\mathrm{RT}}_{f}}\right]$$

that is based on the concept of the fictive temperature T_f_ [16]. T_f_ is defined as the temperature of the undercooled liquid with the same structure as that of the relaxing glass at the given time. The evolution of T_f_ during the structural relaxation depends (see Equations (1) and (2)) on the following quantities: time t, temperature T, relaxation time τ, the universal gas constant R, the apparent activation energy of structural relaxation Δh*, the pre-exponential constant A_TNM_, the non-linearity parameter x (0 < x ≤ 1), and the non-exponentiality parameter β (0 < β ≤ 1). 

Despite the phenomenological nature of the TNM model, various interpretations of its parameters were proposed. The activation energy represents an energetic barrier for the relaxation movements. The pre-exponential factor corresponds to the frequency of relaxation movements. The non-exponentiality parameter reflects the heterogeneity (width of the distribution of the relaxation times), and the non-linearity parameter indicates the degree of interconnectivity and cooperativity between the units carrying the relaxation motions [19,20,21,22]. Consequently, such interpretations can be correlated with the experimentally determined structural changes in the amorphous materials. Compositional trends of the TNM parameters can then be used to obtain generalized information about the structural relaxation behavior within the given glassy system or material family [23,24,25]. Whereas the chemistry-based evolution of the glass transition kinetics absolutely dominates modern scientific research, there is still a considerable amount of papers dealing with the physics-based changes of the structural relaxation motions in the glass transition range. Namely, the deviations between the relaxation kinetics of the deeply relaxed states and in the near-T_g_ equilibrium belong among the current hot topics [26,27,28,29,30]. Another even more extensive field of research is focused on the plasticization-induced changes in the glass transition behavior [31,32,33,34,35]. It is thus quite surprising that similar attention has not been paid to the relaxation movements being (possibly) influenced by the presence of the adjacent crystalline phase. 

Formation of the crystalline phase in amorphous materials is usually considered undesirable, as the nuclei and/or crystallites can significantly change the workability and many other key properties of glassy matrices. However, there are a number of important applications where the co-existence of the amorphous and crystalline phases is crucial/unavoidable. For example, in the glass–ceramics, a controlled formation of crystallites within the amorphous matrix is utilized to improve the mechanical properties [36,37,38]. Numerous polymers are also semi-crystalline in nature, where a single polymeric chain can be part of a crystalline segment as well as of an amorphous domain [39,40,41]. So far, the absolute majority of the (few) papers dealing with the influence of the crystalline phase on the relaxation processes was performed for polymeric materials. The effect of ~25 w.% crystallinity on the structural relaxation of poly(L-lactic) acid was studied in [42]—the following changes in the TNM parameters were observed with the presence of the crystalline content: Δh* ≈ 1049 → 901 kJ·mol^−1^, x ≈ 0.10 → 0.12, β ≈ 0.40 → 0.35, no change in A. These findings indicate that the mobility of the amorphous phase confined between the crystalline lamellae is dynamically distinct from that of the bulk amorphous. Also, the amorphous chains in the close vicinity of the crystalline regions will eventually undergo conformational rearrangement at higher temperatures, increasing the dynamic heterogeneity and broadening the distribution of relaxation times [42]. In protein-based thermoplastics [43,44], the presence of crystalline content constrains the motions of amorphous chains, giving rise to a broader distribution of relaxation times (lower β) and an increase in T_g_. In addition, a secondary type of relaxation motion (α_c_ relaxation) can occur for the polymeric chains included in the organized/crystalline structures [43,44]. In the case of thermoplastic polyurethane [45], the increased crystalline content partially suppresses the structural relaxation via restraining the chain motions. This resulted in an increase in T_g_ and a slight increase in β (due to the phase separation of the soft and hard structural segments). Stronger suppression of the relaxation motions occurred for crystallization at low T, where a larger amount of smaller crystalline domains were formed (due to the simultaneously proceeding nucleation and lower growth rate). This resulted in the larger specific area of the crystalline/amorphous interface. Conforming findings were also observed for the poly(L-lactic) acid in [46], where the relaxation-hindering effect of crystallinity was confirmed to be only slightly below T_g_. At these temperatures, the segmental movements are still allowed, and the dynamics of the mobile amorphous phase are significantly affected by the crystalline microstructure. On the other hand, well below T_g_, only local movements occur in both the mobile and rigid amorphous phases, and the effect of imposed crystallinity is minor. The effect of crystallinity on the structural relaxation of poly(p-dioxanone) was studied in [47]. With the increasing amount of crystalline content, Δh* and A remained unchanged, β suddenly and largely decreased (0.50 → 0.15) only at a very high degree of crystallinity, and x continually increased from 0.45 to 0.80. A slow low-T formation of the crystalline phase in the inorganic polymeric glass Se_70_Te_30_ [48] led to an increase in Δh* from 308 to 366 kJ·mol^−1^. 

Contrary to the majority of the existing literature, in the present paper, the effect of crystallinity on structural relaxation will be studied for a low-molecular glass—the amorphous indomethacin (IMC). Indomethacin is a nonsteroidal anti-inflammatory drug that is used for various medical conditions. It has analgesic, antipyretic, and anti-inflammatory properties [49,50,51,52]. One of the main uses of indomethacin is in the treatment of pain and inflammation associated with conditions such as arthritis, gout, and ankylosing spondylitis. It is also used to relieve pain and reduce inflammation after surgery or injury [49]. Indomethacin has been found to be effective in the treatment of certain types of headaches, such as paroxysmal hemicrania and hemicrania continua [50]. In contrast to polymeric materials, where the crystalline phase is formed within the material volume, the low-molecular organic glasses preferentially crystallize from the surface. Note that the preferential surface crystal growth is a consequence of these glasses having very high surface mobility/diffusion and low molecular weight [53,54,55]. A highly energetic crystallization center (such as a defect or a micro-crack) is, however, still needed for the crystal growth to proceed [53,54,55]. Hence, the surface crystalline content should theoretically have zero impact on the structural relaxation in bulk material. It should also increase with the decreasing particle size of the powdered glass, where the crystal growth propagating along the micro-cracks and other mechanically induced defects can start to constrain the molecular relaxation motions. These hypotheses will be tested in the present paper, using the calorimetric measurements of enthalpy relaxation for various IMC powders (differing size-wise) crystallized to a gradually increasing extent.

## 2. Results

**Figure 1:**
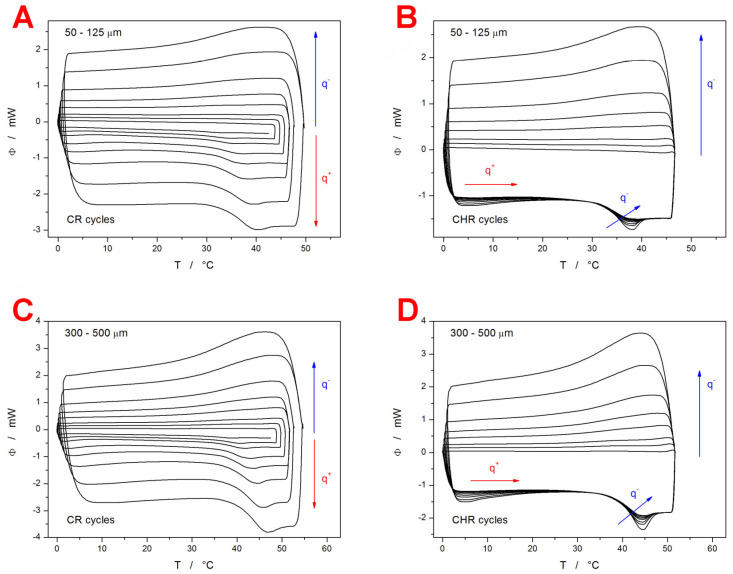
Sets of CR (graphs **A**,**C**) and CHR cycles (graphs **B**,**D**) obtained for the IMC powders with particle sizes 50–125 µm (graphs (**A**,**B**) and 300–500 µm (graphs **C**,**D**). The exothermic effects evolve in the upward direction. In the case of CR cycles, the arrows and symbols q^−^ and q^+^ denote the parts of the DSC data in which the cooling and heating steps (respectively) of the CR cycles are shown. Absolute magnitudes of q^−^ and q^+^ being applied in the corresponding steps of the cyclic program increase in the directions of the given arrows. In the CHR measurements, q^−^ varied, and q^+^ was always 10 °C·min^−1^. The arrow and symbol q^−^ denote the parts of the DSC data that differ in accordance with applied q^−^. In the upper part of the graph (where cooling steps are depicted), the absolute magnitude of q^−^ increases with the arrow. In the part where the heating steps are shown, the arrow denotes the increase in |q^−^| in the preceding cooling step.

Differential scanning calorimetry (DSC) was used to perform two types of cyclic relaxation experiments—the constant ratio (CR) cycles (heating rate q^+^ similar to the rate of cooling q^−^ in the preceding step) [56] and the constant heating rate (CHR) cycles [57]. The experiments were performed for two batches of the as-prepared powdered IMC (fine powder with particle size 50–125 µm and coarse powder with particle size 300–500 µm). The DSC curves obtained within these measurements are displayed in Figure 1. 

The most prominent feature is the significant decrease in T_g_ (by ~6 °C) for the fine IMC powder in comparison with the coarse particle size fraction. Since this ΔT_g_ difference is preserved unchanged even at the lowest heating rates (q^+^), the influence of the thermal gradients within the samples or during the heat transfer to the DSC sensor can be ruled out [56]. Note the difference between the larger compact grains vs. a multilayer of smaller grains. The former has worse contact with the bottom of the DSC pan. The latter has better contact with the bottom of the DSC pan but also has air gaps in between the individual IMC grains stacked on top of each other. As will be shown later in this section, the initial powdering of the IMC bulk glass was not associated with any formation of crystalline phase, nor did the increase in the sample surface lead to increased adsorption of water from the atmosphere (which could have been an additional reason for the T_g_ decrease). This leaves only the increased surface area and the increased presence of mechanically induced defects as the potential reasons for the decrease in T_g_ of the fine IMC powder. The surface of the low-molecular organic glasses (IMC included) is known to exhibit faster self-diffusion by an order of magnitude, which can lead to the so-called glass-crystal growth [58,59] along the micro-cracks. This effect is, however, strictly surface-located and proceeds below T_g_. As such, it should not considerably influence the structural relaxation processes that proceed within the materials volume. On the other hand, the presence of mechanical defects (edges, micro-cracks, dislocations, surface corrugation,…) can certainly induce changes in the bulk material behavior [60]. However, these changes should probably still propagate only in the vicinity of these defects, and the majority of the material’s volume should remain uninfluenced. Moreover, the mechanical defects and grain boundaries are usually perceived as barriers to diffusion [61,62], which should lead to an increase in T_g_. A possible explanation for the lowered T_g_ may be based on an increased release of the internal stresses (originating from the melt-quench procedure) [63,64] during the more intense grinding/powdering. The existence of these stresses was confirmed experimentally during the powdering process, where even light tapping led to the shattering of the larger IMC grains. Note that for the low-molecular organic glasses, annealing below/at T_g_ (which is normally used in the glass industry to release internal stress) is not a good option due to the fast degradation of the amorphous structure via the glass-crystal growth that proceeds at maximum rate at these temperatures.

**Figure 2:**
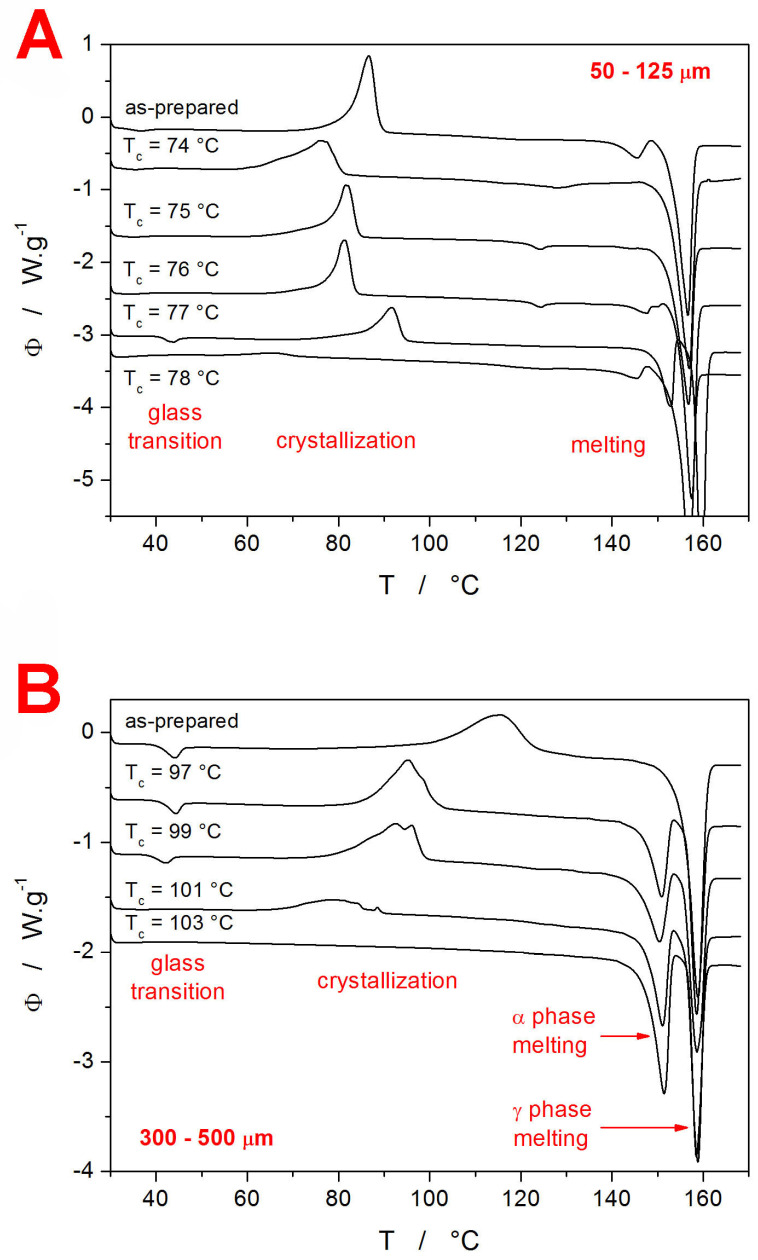
Sets of DSC curves obtained for the amorphous and partially crystallized IMC powders after their full experimental exploitation (performed CR and CHR cyclic measurements). The exothermic effects evolve in the upward direction. Temperature ranges for the most important thermo-kinetic phenomena are indicated. The zoomed-in glass transition and crystallization regions are shown in the Supplementary Materials. Graphs (**A**,**B**) show the data for the 50–125 µm and 300–500 µm powders, respectively.

For each of the two amorphous IMC powders, a series of samples with different contents of crystalline phase was prepared by heating the material in DSC up to a (gradually increasing) selected temperature T_c_ within the crystallization range. In theory, the achieved degree of the crystalline content should be determinable directly from the corresponding DSC curves measured during the preparation phase. However, the slight delay between the heating and the consequent heating step, as well as the finite rate of the cooling, introduce too large errors in the calculation. Therefore, the amount of the crystalline phase within the samples was determined indirectly by heating the fully processed samples (after performing the intended sets of CR and CHR cycles) up to the melting point—see Figure 2. From these DSC records, the degree of crystalline content χ_c_ can be calculated as:

(3) $$\chi_{c}=1-\frac{\Delta H_{c}\cdot {\Delta H}_{m}^{A}}{{\Delta H}_{c}^{A}\cdot \Delta H_{m}}$$

In Equation (3), ΔH_c_ and ΔH_m_ are the enthalpies associated with the crystallization and melting processes, and the superscript “A” denotes the values obtained during the heating of the fully amorphous (as-prepared IMC powder). Apart from the T_g_-related information (which will be introduced in detail later in this section), the DSC curves from Figure 2 also provide a very interesting insight into the crystallization behavior of amorphous and partially crystalline IMC. The coarse powder (300–500 µm) shows a relatively common behavior consistent with previous research conducted in this field [20,65]—i.e., the presence of the low-T-crystallized α polymorphic phase accelerates the crystal growth during the consequent secondary crystallization step (depicted in Figure 2B). On the contrary, in the case of the finely ground IMC powder (Figure 2A), the development of the crystallization behavior with the increasing content of the initially present crystalline phase is rather complex. In particular, a non-monotonous dependence of the crystallization temperature on T_c_ (and, by translation, on χ_c_) emerges. In addition, the 50–125 µm DSC data show a small melting peak at ~123 °C. This may be an indication of the unstable δ IMC polymorph (characteristic melting temperature T_m_ = 129 °C [66]) usually prepared only from a solution, but it may also be possible evidence for an entirely new IMC polymorph. Either way, the double-step crystallization of IMC shows very interesting and unusual data and appears to be worth pursuing in the future.

**Figure 3:**
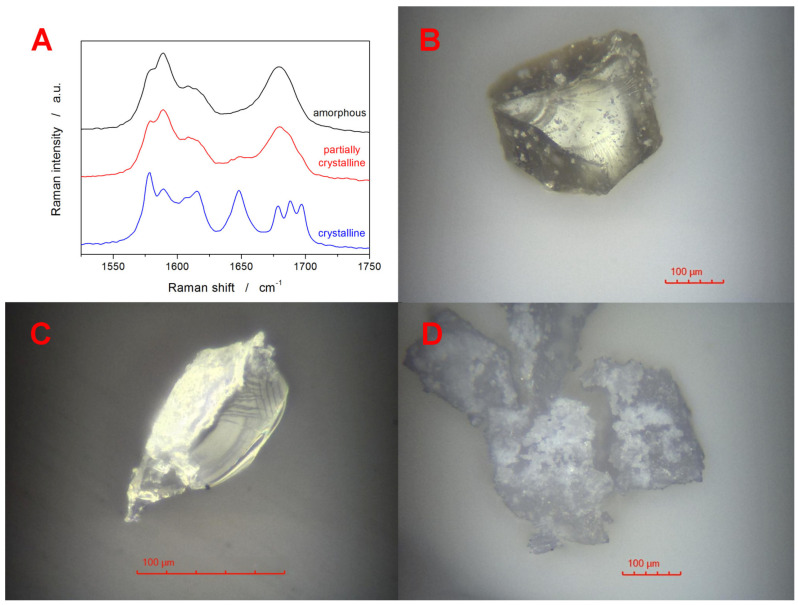
(**A**) Raman spectra for the amorphous, partially crystalline, and fully crystalline IMC powders. (**B**) Fully amorphous IMC grain. (**C**) Fragment of a broken partially crystallized IMC grain. (**D**) Fully crystalline IMC grains.

The fully amorphous as well as partially crystallized IMC powders were further characterized by means of Raman and optical microscopies. The Raman spectra depicted in Figure 3A show the spectral range in which the vibrational differences between the amorphous and crystalline IMC samples can be most easily recognized. Whereas the amorphous IMC is characterized by the broad Raman band at 1685 cm^−1^, γ-IMC is characterized by the 1700 cm^−1^ band (benzoyl C=O stretching), and α-IMC is characterized by bands at 1650 (benzoyl C=O stretching), 1680 (benzoyl C=O stretching), and 1692 cm^−1^ (acid O-C=O stretching) [66,67]. Note that the partially crystalline sample was obtained by heating the IMC powder just below the onset of the DSC crystallization peak, i.e., no exothermic signal was yet observed on the DSC curve. Nonetheless, the Raman spectrum already shows a slight small band at 1650 cm^−1^ and a shoulder near 1700 cm^−1^, confirming that both polymorphs form under these conditions simultaneously. The multicomponent analysis (performed in the OMNIC Specta 2.1 software from ThermoFisher Scientific, Waltham, MA, USA) of the partially crystallized sample determined 14 % crystallinity, which is significantly more than would be expected based on the DSC data. However, it has to be borne in mind that the Raman microscopy is a surface-sensitive technique, with the signal collection being performed from a sphere with ~10–15 µm diameter. In the case of the fully DSC-crystallized IMC sample, all crystalline bands (1650, 1680, and 1700 cm^−1^) are well developed. Their ratio indicates that the α-polymorph is significantly more represented—the sole γ-IMC would exhibit the 1700 cm^−1^ band with ~triple the intensity of the present signal within the crystalline spectrum. The corresponding optical micrographs obtained in the reflective mode for the samples from Figure 3A are shown in Figure 3B–D. The fully amorphous sample shows the typical glassy fractures and is covered with occasional IMC dust particles. Note that the amorphous nature of these formations/aggregates was indeed confirmed via Raman microscopy. The particle in Figure 3C shows a fragment of a broken partially crystallized IMC grain. The right side of the particle corresponds to the interior glassy fracture, and the left side shows the top-side view of the surface crystalline layer (thickness of ~4–5 µm). The IMC grains in Figure 3D show fully crystallized samples. After breaking these samples, a fine powder with essentially the same surface morphology was formed, confirming that the whole of the inner volume was fully crystalline.

**Figure 4:**
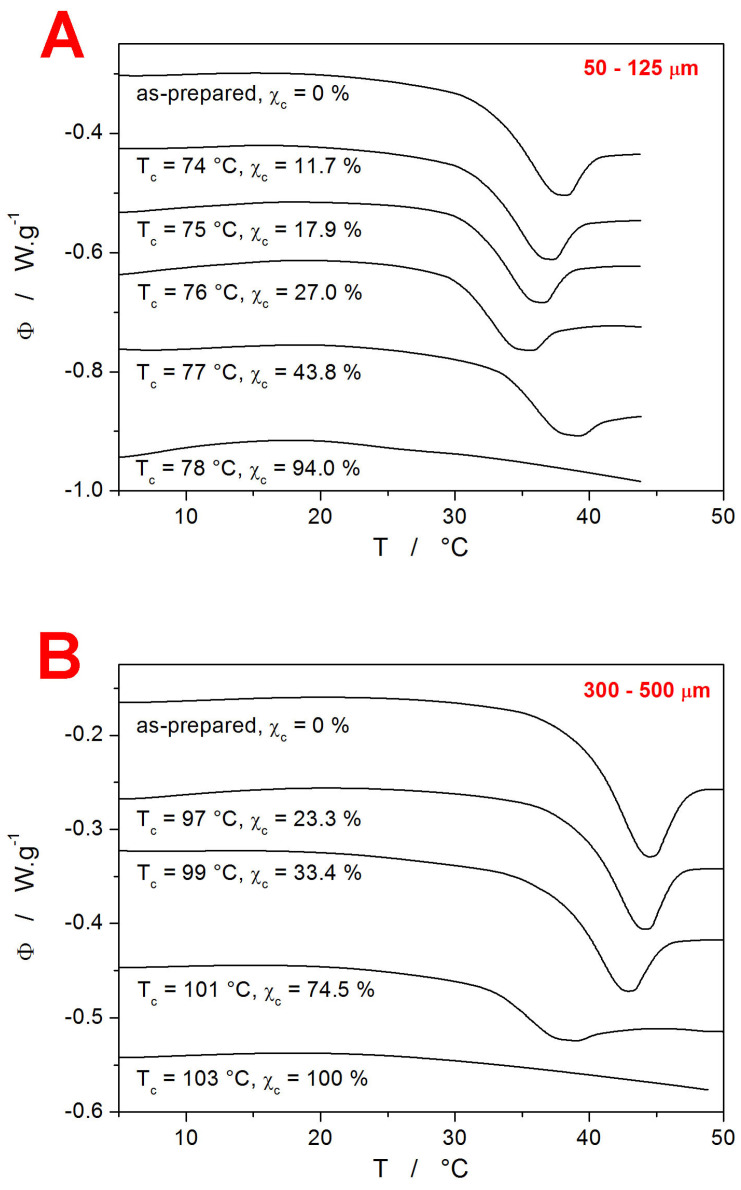
DSC curves obtained within the last cycles (q^−^ = 1 °C∙min^−1^ & q^+^ = 10 °C∙min^−1^) of the CHR experiments performed for the amorphous and partially crystalline IMC powders. The exothermic effects evolve in the upward direction. The degree of crystallinity is indicated by χ_c_ and by the corresponding T_c_s. Graphs (**A**,**B**) show the data for the 50–125 µm and 300–500 µm powders, respectively.

Each partially crystallized IMC sample was subject to a set of CR and CHR relaxation cycles. The evolution of the DSC signal in the glass transition range (displayed for each sample via the q^−^ = 1 °C∙min^−1^ & q^+^ = 10 °C∙min^−1^ CHR cycle) with increasing T_c_ is shown in Figure 4. 

Interestingly, the T_g_ exhibits similar evolution with T_c_ and particle size to the positions of the crystallization peak in Figure 2—a monotonous decrease for the 300–500 µm powder and a decrease followed by an increase at the high degree of crystalline content for the 50–125 µm powder. Thus, the presence of mechanical defects and the consequent distribution of the crystalline phase throughout the glassy IMC matrix (the crystallites grow preferentially along the micro-cracks, both on the surface and inside the bulk material) play a pivotal role in the structural relaxation processes of the low-molecular glasses as well. In particular, two competing phenomena appear to be influencing the T_g_ position. First, a similar effect to that described above regarding the shift of T_g_ with particle size may also be responsible for the decrease in T_g_ with the increasing degree of the crystalline content. Since the T_g_s of both powder fractions are still spaced apart significantly even after heating to relatively high temperatures (76 and 99 °C, respectively), the increased temperature itself evidently does not play a major role in self-healing and/or releasing the stress within the material. The increased material mobility thus has to be directly connected to the presence of the crystalline phase, which grows at the most stress-exposed locations—micro-cracks and surfaces. In the case of the 50–125 µm powder, a significant increase in T_g_ was observed (see Figure 4A) at a higher degree of crystallinity. This is consistent with numerous literature reports for polymeric materials, where the immobile crystalline phase restricts the segmental and chain relaxation motions [42,43,44,45,46].

**Figure 5:**
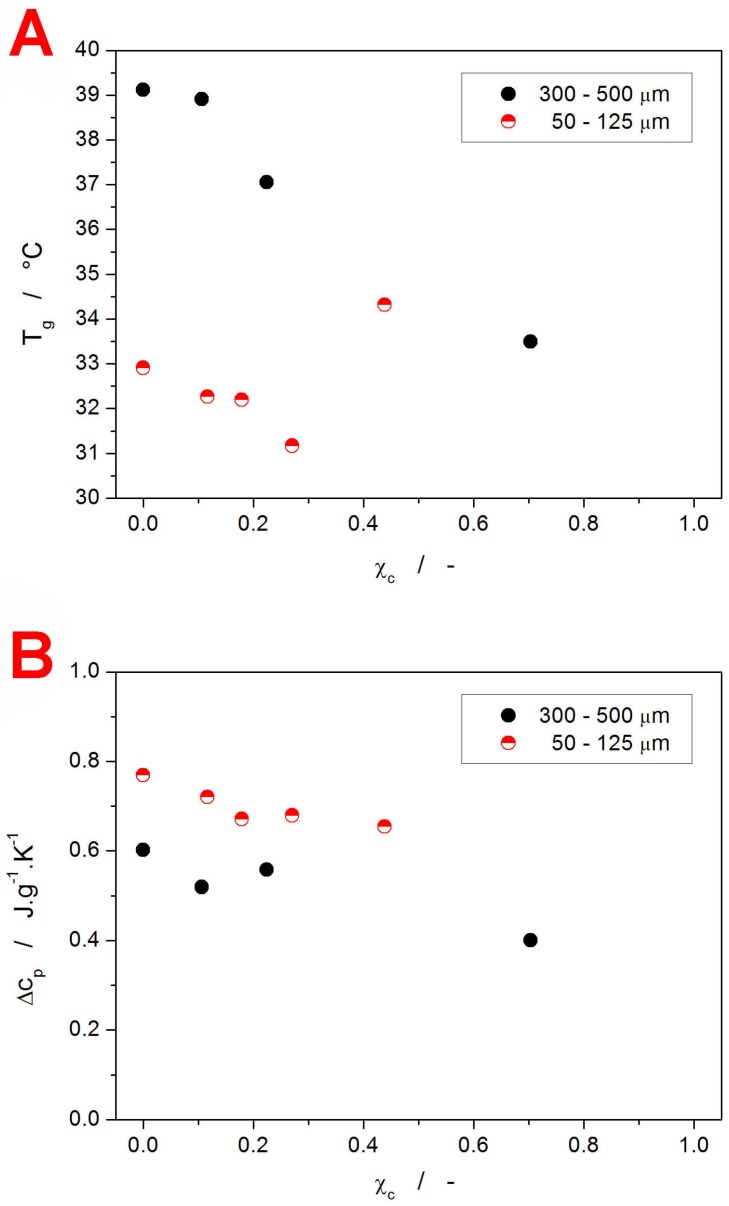
Values of the glass transition temperature T_g_ (graph **A**) and heat capacity difference in the glass transition range Δc_p_ (graph **B**) obtained from the CHR measurements performed for the two studied IMC powders.

The above-introduced qualitative view on the evolution of T_g_ with the degree of crystallinity is quantified in Figure 5A (the T_g_ values were determined from Figure 4; the uncertainties for the T_g_ and χ_c_ determination are approx. 0.5 °C and 0.05, respectively). Apart from the already described features, the graph clearly shows that for the finely powdered material (with the glassy matrix supposedly interwoven with a crystalline network formed along the internal micro-cracks and akin mechanically induced defects), the hindering of the structural relaxation motions occurs relatively early, between 30 and 40% of the crystalline content. On the other hand, for the coarse IMC powder, where the absolute majority of the crystalline phase forms on the grains’ surface [65], no restriction of the structural relaxation (at least in the sense of the decrease in the overall structural mobility expressed by T_g_) occurs even at 70% of crystallinity. Note that it is most probably a coincidence that the increase in T_g_ for the fine powder appears to match (fall onto) the dependence for the coarse powder.

In addition to the T_g_ values, the difference of the undercooled liquid and glass heat capacities Δc_p_ at T_g_ was calculated—see Figure 5B (the uncertainty associated with the Δc_p_ determination is approx. 0.04–0.05 J·g^−1^·K^−1^). Again, the two IMC powders exhibit different base Δc_p_ values. Note, however, that c_p_ is a thermodynamic quantity (as opposed to a kinetically driven one), and as such, the difference cannot be interpreted in the same manner as that of T_g_. Instead, the changes need to be explained in terms of the accessible vibrational modes of the corresponding structures. Assuming that the changes in the chemical contribution to c_p_ (bonding arrangements up to the medium-range structures) are negligible or similar for both types of IMC powder, it is the increase in free volume (configurational entropy) that is responsible for the larger Δc_p_ [68]. Correspondingly, the finely powdered material appears to exhibit a significantly larger free volume (consistent with lower T_g_) as a consequence of either the quenched-in stress being released, or due to the mechanical damage loosening the otherwise compact bulk structure. Whereas the decrease in Δc_p_ with χ_c_ follows the generally accepted concept of the amount of the amorphous phase being proportional to the Δc_p_ value, quantitatively, significantly larger apparent Δc_p_ values were obtained. A possible explanation might involve some new vibrational modes arising either from within the crystalline phase alone or from the amorphous/crystalline interfaces. Nonetheless, further research is definitely needed in this regard.

## 3. Discussion

**Figure 6:**
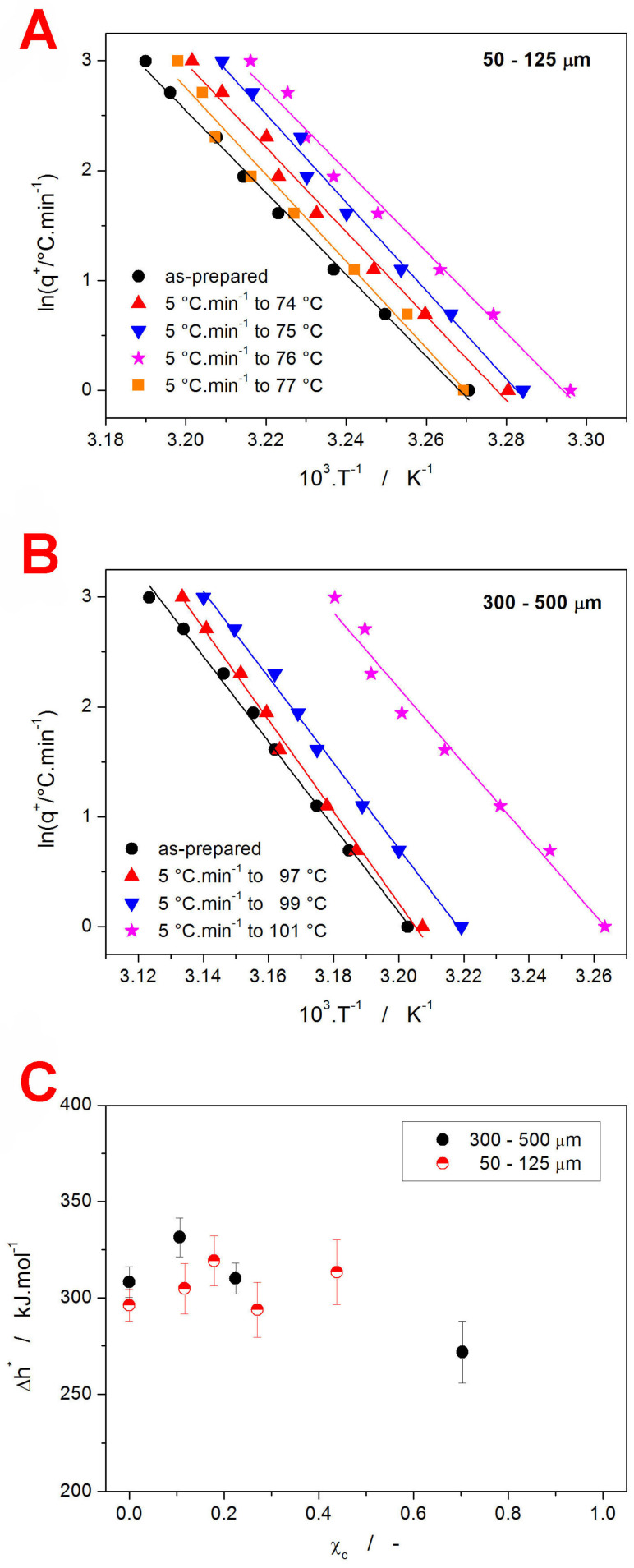
(**A**) The evaluation of Δh* according to Equation (4) from the CR cycles measured for the amorphous and partially crystallized 50–125 µm IMC powders. (**B**) The evaluation of Δh* according to Equation (4) from the CR cycles measured for the amorphous and partially crystallized 300–500 µm IMC powders. (**C**) The Δh* values determined in accordance with Equation (4) and corrected by applying Equation (5) for the amorphous and partially crystallized IMC powders. The values are displayed in dependence on the degree of crystallinity χ_c_.

Although several very interesting features of the crystallinity-influenced glass transition behavior of the powdered IMC were introduced in the previous section, true insight into the structural relaxation kinetics can be gained only by a rigorous mathematical description of the experimental data. In the present case, the enumeration of the TNM model equations (Equations (1) and (2)) will be used to determine the evolution of the relaxation kinetics with χ_c_. Following the guide [69] for the evaluation of the DSC relaxation data, the apparent activation energy of structural relaxation Δh* needs to be determined first. In this regard, the most robust and reliable solution is the evaluation from the CR cycles based on the following equation:

(4) $$-\frac{{\Delta h}^{*}}{R}={\left[\frac{\mathrm{dln}\left|q^{-}\right|}{d\left(1/T_{P}\right)}\right]}_{q^{-}/q^{+}=\mathrm{const}}$$

where T_p_ is the temperature corresponding to the maximum of the relaxation peak/overshoot. These dependencies are for the present amorphous and partially crystalline IMC powders shown in Figure 6A,B.

Apart from the confirmation of the T_g_ being shifted with χ_c_ in the same manner in the whole q^+^ range, the linearity of the obtained dependences also unambiguously rules out any significant influence of the thermal gradients within the samples/system [56]. As was shown in [70], Equation (4) systematically overestimates the apparent activation energy, and the following correction needs to be applied to calculate the true Δh* values:

(5) $$\left[\left(\Delta h_{\exp}^{*}/R\right)-\left(\Delta h_{\mathrm{true}}^{*}/R\right)\right]\cdot 100\%=4.218\cdot 10^{-5}{\left(\Delta h_{\mathrm{true}}^{*}/R\right)}^{2}+4.841\cdot 10^{-2}\left(\Delta h_{\mathrm{true}}^{*}/R\right)+\left[9.885\cdot 10^{1}/\left(\left(\Delta h_{\mathrm{true}}^{*}/R\right)-1.276\right)\right]$$

where the indices “exp” and “true” denote the experimentally obtained (via Equation (4)) and true values of activation energy. In the present case, the magnitude of the Δh* corrections ranged between 4.3 and 4.6%. The χ_c_ dependences of the activation energy are shown in Figure 6C. In their absolute magnitude, the Δh* values comply with the typical Δh* range reported for the low-temperature organic and inorganic glasses. With the increasing degree of crystalline content, the activation energy of IMC structural relaxation remains roughly constant up to χ_c_ ≈ 0.5 and appears to slightly decrease (by ~10%) at higher χ_c_. Note that the preparation of IMC powders with 1 > χ_c_ > 0.5, where the glass transition phenomenon would manifest clearly enough, was found to be extremely difficult, hence the low amount of data in the corresponding χ_c_ range. As was already stated in the introductory part, various Δh*-χ_c_ trends exist in different materials [42,47,48]. Regarding the interpretation of the data shown in Figure 6C, the constancy of Δh* in the low-χ_c_ range implies that similar explanations cannot be used for the evolution of T_g_ and Δh*. The increasing content of the crystalline phase (existing either on the surface of the bulk material or being permeated throughout the amorphous phase) seems to have no significant influence over the number of the inter- or intra-molecular bonds that need to be broken during the relaxation movements. 

The pre-exponential factor A_TNM_ (incorporated in the TNM model—see Equation (2)) was for the individual amorphous and partially crystalline IMC samples determined by means of curve-fitting based on the non-linear optimization [71]. The corresponding lnA_TNM_ values are for the present IMC samples listed in Table 1. 

The curve-fitting was, however, not of sufficient quality for the reliable determination of the TNM parameters β and x (divergence to physically senseless values due to the instrumentally distorted asymmetry of the relaxation peak). On the other hand, with the knowledge of Δh* and A_TNM_, a robust evaluation in terms of the simulation-comparative method [72] can be used even for such data to accurately extract the TNM kinetic information. The method utilizes the comparison of the experimental and theoretically simulated dependences of the height of the normalized relaxation peak C_p_^max^ during the CHR cyclic experiments. The normalization of the relaxation data is based on the following equation:

(6) $$C_{p}^{N}\left(T\right)=\frac{C_{p}\left(T\right)-C_{\mathrm{pg}}\left(T\right)}{C_{\mathrm{pl}}\left(T\right)-C_{\mathrm{pg}}\left(T\right)}$$

In Equation (6), C_p_^N^(T) is the normalized relaxation signal, C_p_(T) is the measured signal, and C_pg_(T) and C_pl_(T) are the extrapolated DSC signals in the glassy and undercooled liquid regions, respectively. 

**Figure 7:**
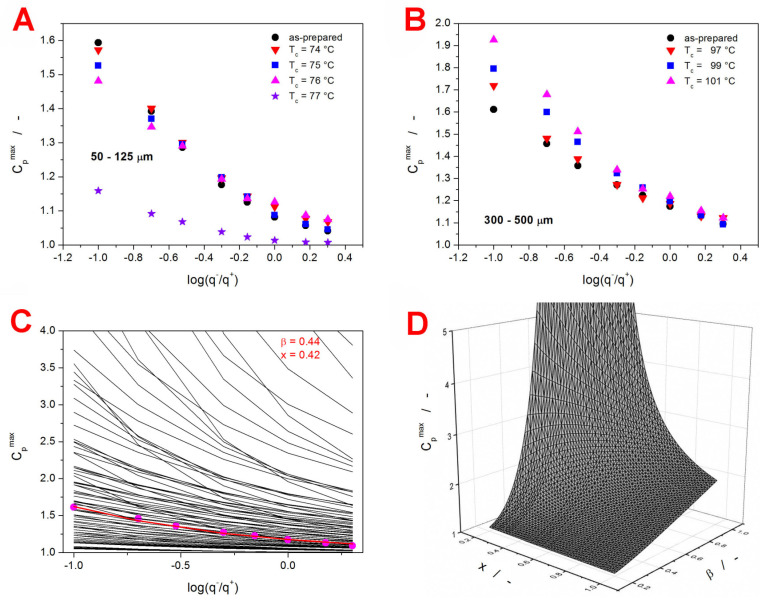
(**A**) C_p_^max^ values determined from the CHR cycles measured for the amorphous and partially crystallized 50–125 µm IMC powders. (**B**) C_p_^max^ values determined from the CHR cycles measured for the amorphous and partially crystallized 300–500 µm IMC powders. (**C**) Example comparison of the experimental C_p_^max^ values determined from the CHR cycles measured for the amorphous 300–500 µm IMC powder (points) and of the C_p_^max^-log(q^−^/q^+^) dependences theoretically simulated for the corresponding temperature history and Δh* and A_TNM_ parameters (lines). The red line and the shown β and x values indicate the best correspondence of the theoretically simulated data with the experimental data. The colored version of this graph is included in the Supplementary Materials. (**D**) Example of the 3-dimensional hyperspace extracted from graph C for log(q^−^/q^+^) = 0.

The dependences of C_p_^max^ on the glassy state thermal history achieved during the given CHR cycle are for the present IMC samples shown in Figure 7A,B.

Although the C_p_^max^-log(q^−^/q^+^) dependences exhibit rather small changes with increasing crystallinity, clear trends can be recognized. As the content of the crystalline phase increases, the 50–125 µm dependences decrease in slope, and the slope of the 300–500 µm dependences increases. To each IMC sample (defined by the given Δh* and A_TNM_ combination), a unique series of 6561 theoretically simulated C_p_^max^-log(q^−^/q^+^) dependences was attributed based on the correspondence of the Δh* and A_TNM_ values. Each such database contained dependences simulated for the defined Δh* and A_TNM_ combination. Hence, the Δh* and A_TNM_ were fixed, while the β and x parameters varied in the 0.2–1.0 range with the step of 0.01, therefore resulting in 6561 unique TNM parameters combinations. From each database, the theoretically simulated C_p_^max^-log(q^−^/q^+^) dependence closest to the corresponding set of the experimental values was chosen numerically, and the matching combination of the β and x parameters was obtained. An example visual representation of this procedure is depicted in Figure 7C. Note that the figure displays only every tenth theoretically simulated dependence (β and x changing with step of 0.1). In practice, the grid of theoretically simulated data was 10 times denser. To better visualize the course of the individual dependences, a vertical “cut” (at log(q^−^/q^+^) = 0) through the data from Figure 7C is displayed as a 3D plot in Figure 7D. In particular, Figure 7D shows the simulated dependence of C_p_^max^ on β and x for q^−^ = 10 °C·min^−1^. Essentially, each experimentally determined C_p_^max^ value was compared with one such hyperspace. This process was performed simultaneously for the C_p_^max^ values corresponding to the given series of the CHR cycles, with the least sum of squared residue being the decisive metric. 

**Figure 8:**
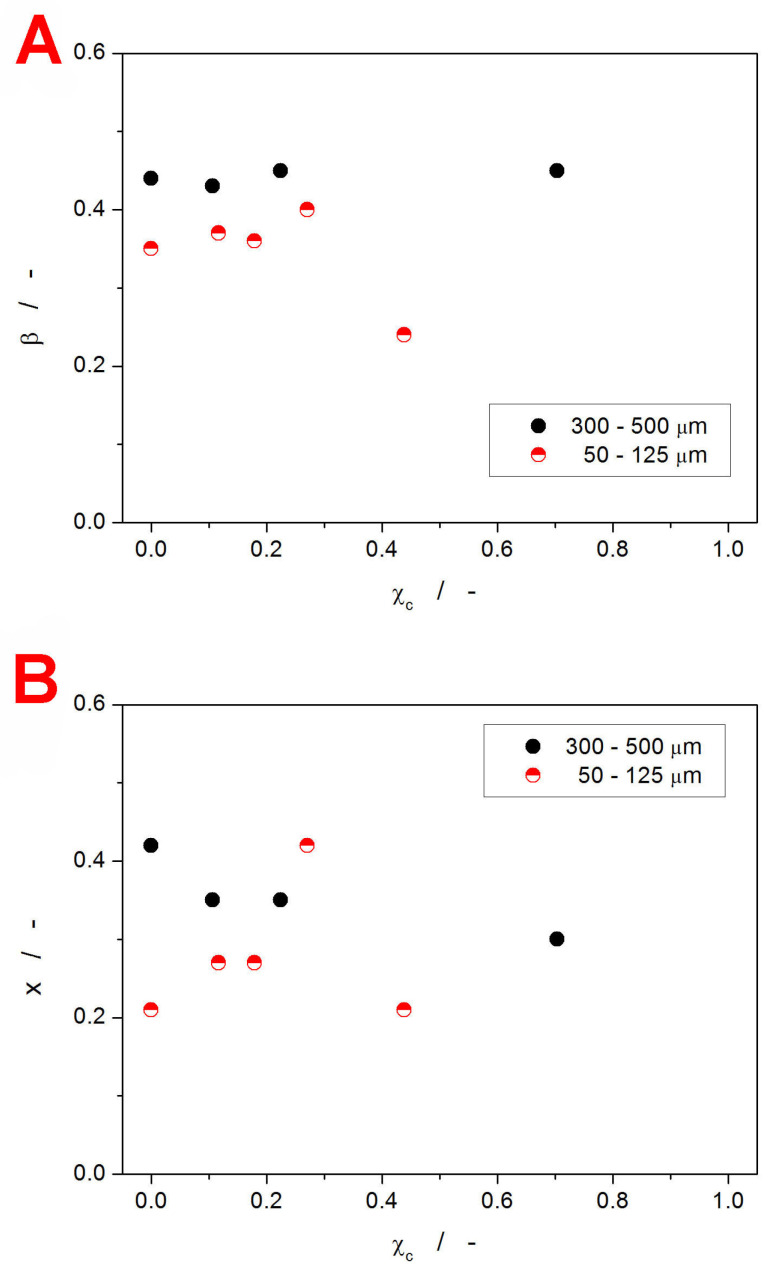
The β (graph **A**) and x (graph **B**) values determined by means of the simulation-comparative method from the CHR cycles measured for the amorphous and partially crystallized IMC powders. The values are displayed in dependence on the degree of crystallinity χ_c_.

The values of the β and x parameters determined by the simulation-comparative method [72] are shown in Figure 8. 

Both TNM parameters were determined with the ~±0.02 errors. Starting with the non-exponentiality parameter β, the crystalline layer formed preferentially on the surface of the amorphous grains (the case of the 300–500 µm particle size fraction) does not influence the degree of heterogeneity within the amorphous matrix. On the other hand, in the case of the 50–125 µm powder, the uniformity of structural motions employed in the structural relaxation slightly increases with χ_c_, but above χ_c_ ≈ 0.3, the distribution of the relaxation times significantly broadens (as β decreases). This appears to correlate well with the evolution of T_g_ with χ_c_. Consequently, above a certain crystallinity threshold, the amorphous phase in the close vicinity of the glass/crystal interface undergoes conformational rearrangement, forming larger super-structures (with the changes possibly induced by a process akin to templating at the crystal–crystal interface). This assumption is further supported by the trends in the x-χ_c_ dependence (see Figure 8B), where the sharp decrease observed for the 50–125 µm powder above ~χ_c_ = 0.3 can be interpreted [20,47] as an increase in the interconnectivity of the relaxing structure. Note that the decrease in the non-linearity parameter x indicates a larger dependence of the relaxation motions on the actual material’s structure (as opposed to the dependence on T alone). When comparing these findings to the literature data (introduced in Section 1), the broadened distribution of the relaxation times appears to be quite a common feature [42,43,44,47], also confirmed by our results. The non-monotonic evolution of x with increasing crystalline content was, to the authors’ knowledge, not reported in the literature; the initial steep increase in x can be considered conformant with [47].

## 4. Materials and Methods

The amorphous indomethacin (IMC) was prepared by means of the melt-quench technique from the as-purchased crystalline γ-polymorph (purity > 99%; Sigma-Aldrich, Prague, Czech Republic). The purchased crystalline IMC was melted in a glass vial immersed in an oil bath (heated to 165 °C); the vial was consequently quenched in cold (~10 °C) water. The bulk IMC ingot was then powdered with an agate mortar and pestle and fractionalized using a set of sieves with defined mesh sizes (Retsch, Haan, Germany). In the present study, powder fractions with 50–125 µm and 300–500 µm particle size ranges were used. The powders were stored in the dark in a frozen (−10 °C) desiccator. As the IMC powders were found to nucleate even under these conditions, they were intentionally aged for 7 days so that the number of nuclei was stabilized and the crystallization behavior was as reproducible as possible. 

The initial preparation of the partially crystallized IMC powders, as well as the heat treatment within the cyclic relaxation temperature programs, was realized using the heat flow differential scanning calorimeter DSC Q2000 (TA Instruments, New Castle, DE, USA) equipped with an autosampler, an RCS90 cooling accessory, and T-zero technology. The DSC calibration was performed based on the melting temperatures and enthalpies of In, Zn, and H_2_O standards. All DSC measurements were performed in hermetically sealed Al pans and static air atmosphere; the sample masses were 2.5–3.5 mg (accurately determined to ±0.01 mg). The preparation of the partially crystallized powder samples was conducted based on the preceding test measurements (one for each particle size), performed at 5 °C∙min^−1^ in the 20–180 °C range. Following the exact positions of the crystallization peaks on the temperature axis, a series of crystallization temperatures T_c_ was selected for each powder fraction: for the 50–125 µm powder, the T_c_s of 74, 75, 76, 77, and 78 °C were chosen; for the 300–500 µm powder, the T_c_s were 97, 99, and 101 °C. By heating each sample at 5 °C∙min^−1^ to a selected T_c_, two series of partially crystallized IMC powders were prepared for the two particle size fractions. 

The structural relaxation measurements were based on two types of cyclic temperature programs—the constant ratio (CR) cycles [56] and the constant heating rate (CHR) cycles [57]. In the case of both cycle types, the samples were cyclically cooled and heated through the glass transition region, with the applied cooling rates being q^−^ = 20, 15, 10, 7, 5, 3, 2, and 1 °C∙min^−1^ (in that order). In the CR cycles, the samples were heated at heating rates q^+^ similar in absolute magnitude to the preceding q^−^. The broadest T range (0–55 °C) used for the highest q^+^ was progressively cut on the high-T side for each consequent cycle to limit the risk of unwanted secondary crystal growth, proceeding during the slowest cooling and heating steps to minimum. In the CHR cycles, the samples were always heated at 10 °C∙min^−1^. The maximum temperature limits were 47 and 52 °C for the 50–125 µm and 300–500 µm powders, respectively. For each combination of powder size and T_c_, the set of CR cycles was immediately followed by the set of CHR cycles without removing the pan from the DSC cell to improve the reproducibility. After performing both types of cyclic relaxation measurements, each sample was heated at 5 °C∙min^−1^ in the 30–180 °C temperature range to cause it to be fully crystallized and consequently melted. These final measurements were used to calculate the exact degree of crystalline content formed during the initial preparation of partially crystalline matrices.

In addition to the DSC technique, a Raman microscope DXR2 (Nicolet, Thermo Fisher Scientific, Prague, Czech Republic), equipped with a 785 nm excitation diode laser (30 mW, laser spot size of 1.6 μm) and CCD detector, was used to collect the Raman spectra of the amorphous and DSC-crystallized samples. The experimental setup for the Raman experiments was a 20 mW laser power on the sample, 3 s duration of a single scan, and 50 scans summed in one spectrum. The optical microscope iScope PLMi (Euromex, Arnhem, The Netherlands), equipped with 40× and 80× high-quality objectives and a Moticam visual camera, was used in the polarized reflection mode to check the nature of the typically formed crystallites.

## 5. Conclusions

The influence of the degree of crystallinity χ_c_ on the structural relaxation kinetics (described within the TNM concept) was studied for the coarse (300–500 µm particle size) and fine (50–125 µm) IMC powders. In the case of coarse powder, where the formation of the crystalline phase occurs dominantly on the surface, the glass transition monotonically decreases (by ~6 °C in the 0–70% crystallinity range). Regarding the relaxation dynamics, the activation energy for the relaxation motions and the degree of heterogeneity within the relaxing matrix remain practically unchanged, while the interconnectivity seems to slightly increase with χ_c_. This behavior was primarily explained by the release of the quenched-in stresses (decrease in T_g_) and the consequent slight increase in the structural interconnectivity. For the fine IMC powder, the crystalline phase is assumed to be permeated throughout the bulk glassy matrix to a much higher degree, as it can form along the internal micro-cracks. This results in a significantly distinct behavior, where at higher χ_c_, a sharp increase in T_g_, an increase in interconnectivity, and an increase in the variability of structural units engaged in the relaxation motions occur. This threshold is possibly associated either with the formation of a fully interconnected internal network of crystalline phases (that would significantly constrict the magnitude of the relaxation domains, leading to enforced cooperation of relaxation movements) or with a complete release of internal stresses (that previously constricted the relaxation movements).

Considering the number of surprising findings, consequent research is certainly needed. The following list of questions and research points appears to be the most promising:

- Where is the borderline between the two types of crystal growth penetration into the glassy matrix? Experiments: perform an akin but more extensive study, employing a large number of narrowly defined particle size fractions.
- Is the internal stress truly responsible for the differences in the T_g_ values of the two size-varied powders and for the decrease in T_g_ with χ_c_? Experiments: achieve similar particle size with different levels of stress by gentle tapping, force-based grinding, and, e.g., spray-drying; employ long-term annealing to further differentiate levels of internal stress; correlate T_g_ with the light polariscope results.
- What is the layout of the crystalline phase within the fine partially crystallized glass grains? Experiments: map the layout, e.g., with confocal Raman microscopy.
- Are the findings regarding the evolution of relaxation kinetics universal for other low-molecular organic glasses? Experiments: repeat the study for other materials while focusing on the compounds with the least interfering phenomena, i.e., compounds that exhibit no significantly competing polymorphism and have a zero-to-low rate of glass-crystal growth in the glass transition region.

## Figures and Tables

**Table 1:** Pre-exponential factors from the TNM model determined by curve-fitting for the amorphous and partially crystalline IMC powders.

50–125 µm					
T_c_/°C:	as-prepared	74	75	76	77
ln(A_TNM_/s):	113.5	117.2	123.2	113.6	120.0
300–500 µm					
T_c_/°C:	as-prepared	97	99	101	
ln(A_TNM_/s):	115.5	124.7	117.0	104.2	

## Data Availability

The data are available on request.

## Supplementary Materials

The following supporting information can be downloaded at https://www.mdpi.com/article/10.3390/ijms242216275/s1.
