# Supplementary material for: How the Presence of Crystalline Phase Affects Structural Relaxation in Molecular Liquids: The Case of Amorphous Indomethacin
Source: Int J Mol Sci. 2023 Nov 13;24(22):16275. doi: 10.3390/ijms242216275 (PMC10671508; doi:10.3390/ijms242216275)
Supplement: Supplementary file 1 [file ijms-24-16275-s001.zip › ijms-2718590-supplementary.pdf]

Supplementary Materials

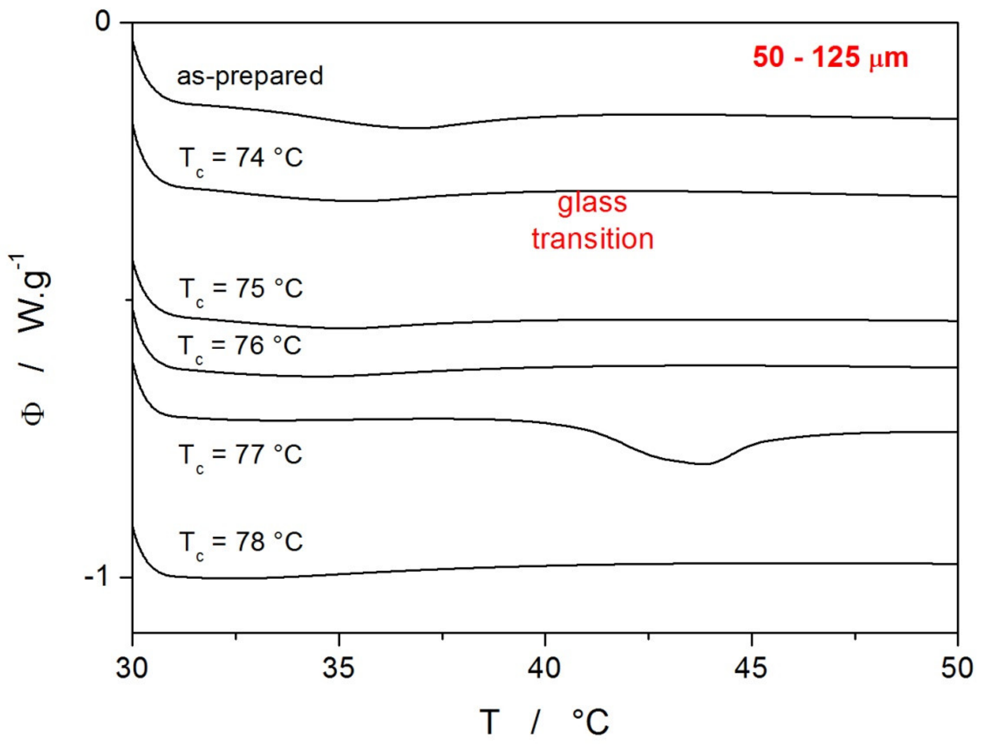

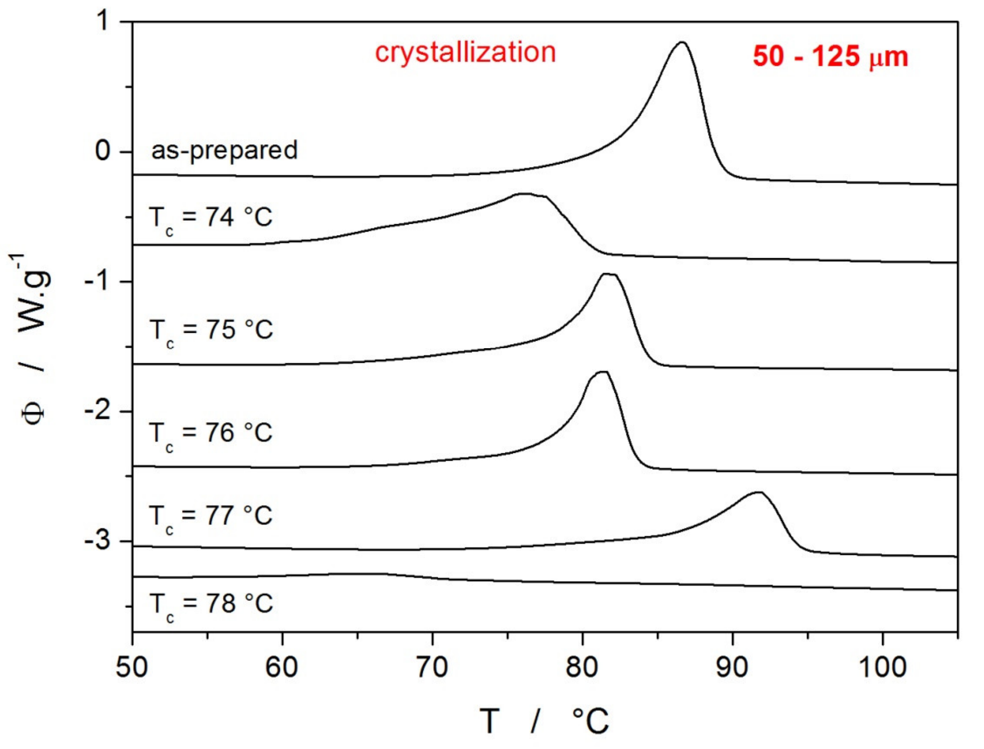

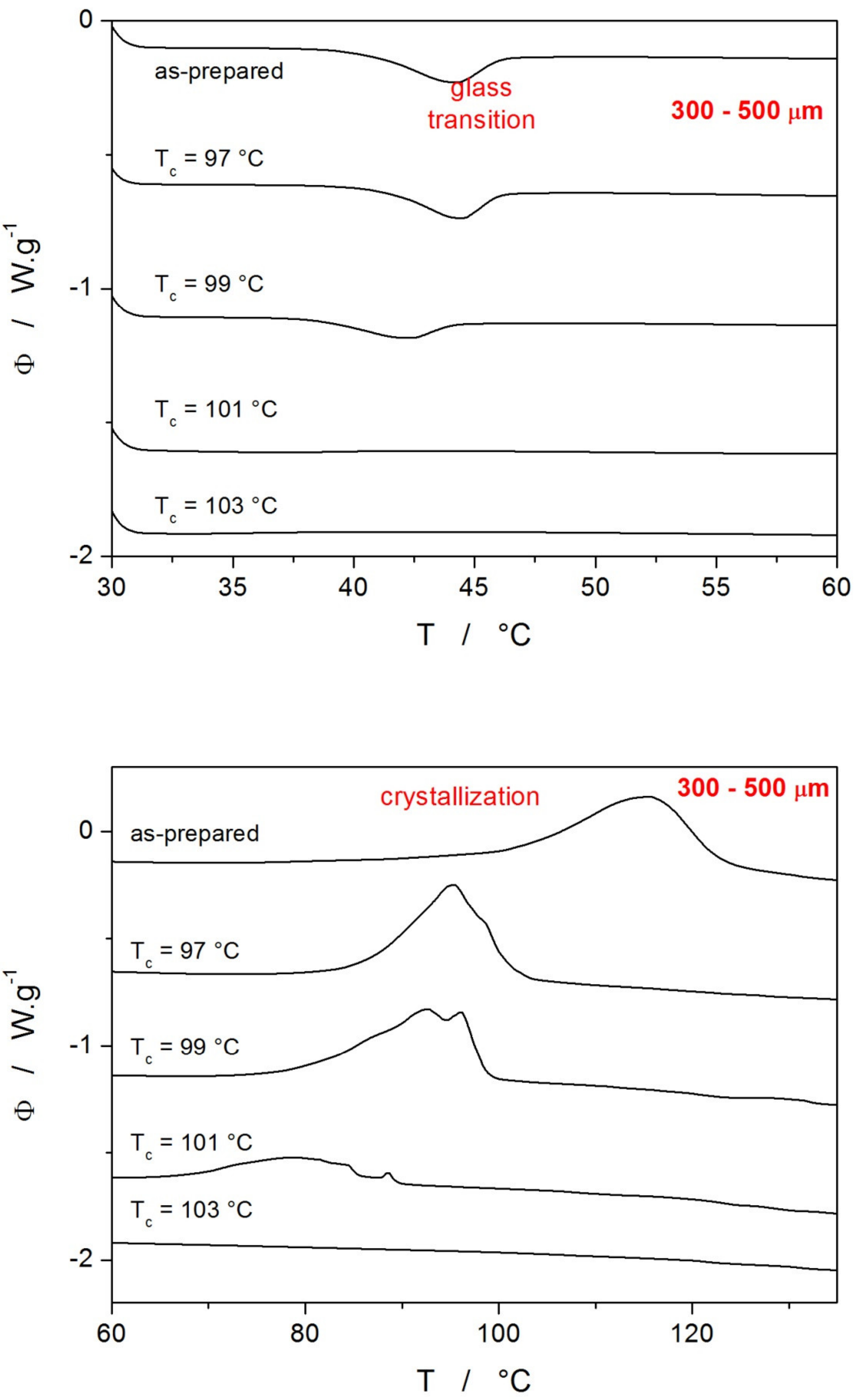

Figure S1. Zoomed in glass transition and crystallization regions from Figure 2.

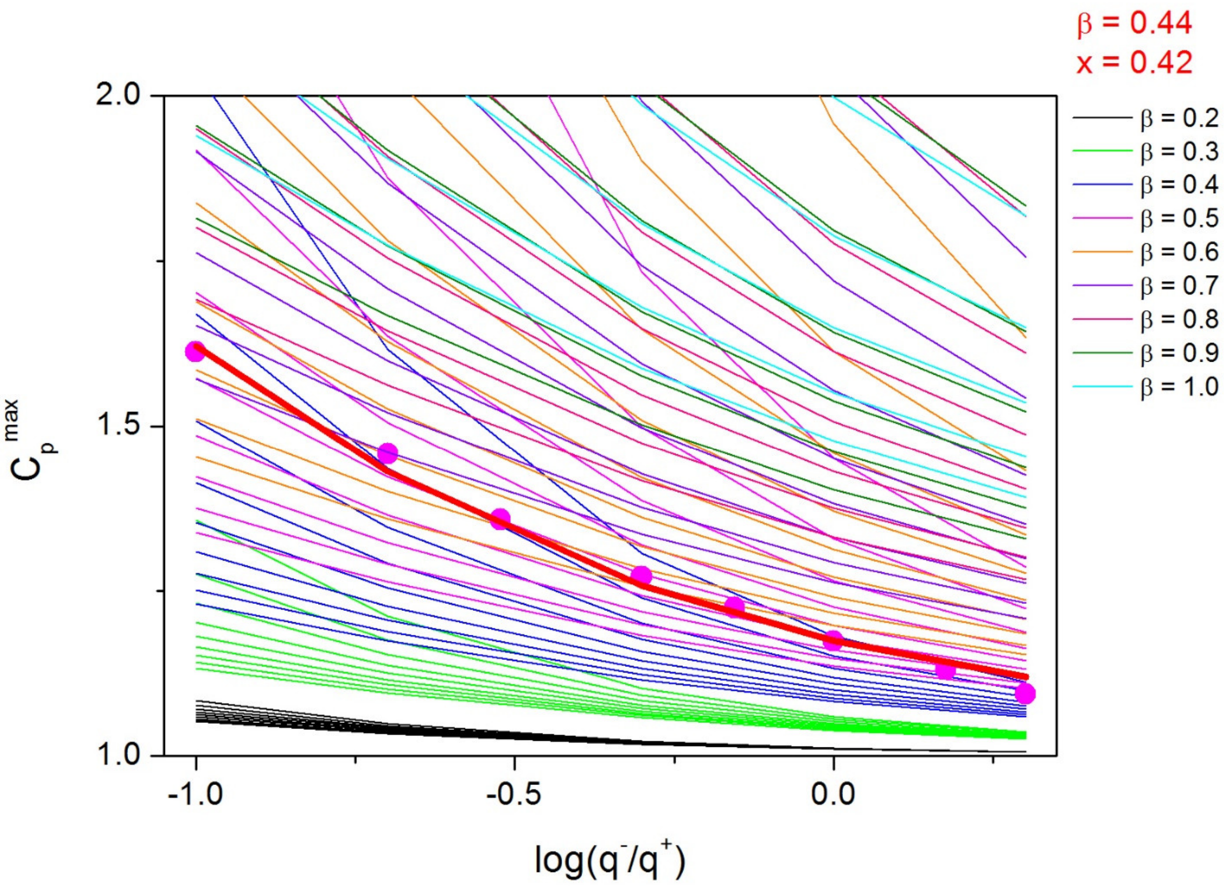

Figure S2. A colored version of Figure 7C. Particular colors correspond to the groups of dependences with similar β value. In each such group, the increasing value of x corresponds to the decrease of C_p^{max}.
